# Central Zika virus infection causes hypothalamic inflammation and persistent insulin resistance in adult mice

**DOI:** 10.1038/s41419-025-08046-5

**Published:** 2025-10-13

**Authors:** Emanuelle V. de Lima, Clara O. Nogueira, Raissa R. Christoff, Emylle Costa-Bartuli, Tathiany Igreja-Silva, Mariana Oliveira Lopes da Silva, Daniel Gavino-Leopoldino, Maria Luiza Móra Santos, Felipe Simões Lemos, Jaderson C. da Costa, Gianina T. Venturin, Iranaia Assunção-Miranda, João M. N. Duarte, Claudia P. Figueiredo, Giselle F. Passos, Julia R. Clarke

**Affiliations:** 1 Faculdade de Farmácia, Universidade Federal do Rio de Janeiro, Rio de Janeiro, RJ Brazil; 2 Instituto de Ciências Biomédicas, Universidade Federal do Rio de Janeiro, Rio de Janeiro, RJ Brazil; 3 Instituto de Microbiologia Paulo de Goes, Universidade Federal do Rio de Janeiro, Rio de Janeiro, RJ Brazil; 4 Instituto do Cérebro, Pontifícia Universidade Católica do Rio Grande do Sul, Porto Alegre, RS Brazil; 5 Department of Experimental Medical Science, Faculty of Medicine, Lund University, Lund, Sweden; 6 Wallenberg Centre for Molecular Medicine, Lund University, Lund, Sweden

**Keywords:** Diseases of the nervous system, Neuroimmunology

## Abstract

Zika virus (ZIKV) is a neurotropic flavivirus capable of infecting the adult brain, however its impact on hypothalamic function and metabolic regulation remains unclear. Here, we show that ZIKV invades the hypothalamus of immunocompetent adult mice, where it replicates and persists, leading to sustained insulin resistance. Following infection, ZIKV RNA and negative strand were detected in the hypothalamus of mice and immunostaining confirmed viral proteins in neurons, especially POMC-positive cells. At 6 dpi, ZIKV induced hypothalamic neuroinflammation, as shown by the upregulation of TNF-α, IL-6, IFN-β, and ISG-15, as well as microglial activation. These inflammatory responses were associated with impaired insulin signaling, characterized by reduced phosphorylation of IRS-1 and S6K, downregulation of insulin receptor mRNA, and decreased anorexigenic response following intracerebral insulin administration. Our data also showed that, despite viral clearance and resolution of hypothalamic inflammation at 30 dpi, reduction in insulin receptor protein levels and hypothalamic insulin resistance persisted. These findings demonstrate that ZIKV replicates in the hypothalamus of immunocompetent adult mice, leading to long-lasting disruption of central insulin signaling. Our study identifies hypothalamic insulin resistance as a novel consequence of ZIKV central nervous system invasion and suggests that viral infections may contribute to long-term metabolic dysfunction, highlighting the need to investigate persistent hypothalamic and metabolic alterations in ZIKV infection-recovered individuals.

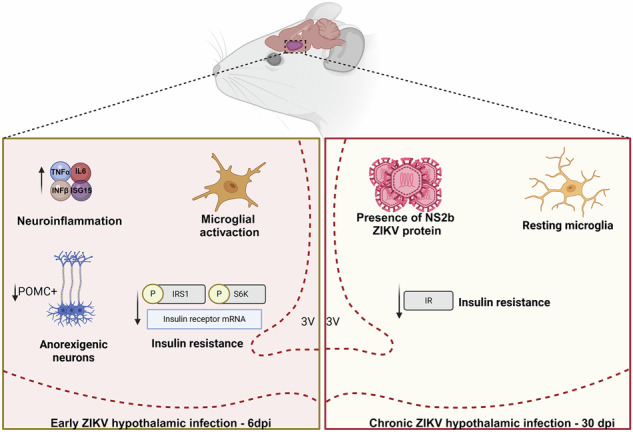

## Introduction

Zika virus (ZIKV) is a neurotropic member of the *flaviviridae* family, known to cause neurological damage in both the developing and mature central nervous system [1, 2]. While early studies focused on describing the consequences of congenital viral exposure, it is now clear that in some adult individuals ZIKV may also invade the central nervous system (CNS), causing acute myelitis, encephalomyelitis, encephalitis, among many others [3–7].

The hypothalamus is a complex structure that plays fundamental roles in controlling various body functions such as temperature, heart rate, hunger, sex behavior, and metabolism [8, 9]. Previous studies have shown that the hypothalamus is targeted by ZIKV, where it may replicate and persist [2, 10, 11]. In a model of postnatal ZIKV infection in suckling mice, which resembles brain viral exposure in the late stages of gestation in humans, a complete and persistent disruption of the hypothalamic-pituitary-gonadal axis was described [10], resulting in impaired thyroid function, limited body growth and delayed testicular development, ultimately impacting fertility [11]. While the hypothalamic impact of ZIKV infection to the mature brain has never been directly addressed, case reports frequently describe manifestation of symptoms strongly suggestive of hypothalamic damage [12–14]. In agreement with the consolidated bidirectional road between inflammation and glucose metabolism homeostasis, previous studies have shown that viral-induced increases in interferon response can directly cause insulin resistance and peripheral metabolic dysfunction [15–17].

We have recently described a mouse model of ZIKV infection and described the mechanisms underlying memory and synaptic damage in the adult brain. We found that the virus reaches its peak in the brains of mice at 6 days post-infection (dpi) and decays by 30 dpi [2]. We also showed that the virus preferentially targets specific brain regions, including cortex, hippocampus, striatum, and hypothalamus [2]. Here, we investigated whether ZIKV CNS infection is associated with changes in insulin signaling specifically in the hypothalamus and whether this ultimately leads to disrupted control of glucose homeostasis. We found that ZIKV reaches the hypothalamus causing increased production of inflammatory mediators, microglial activation and persistent disruption of insulin signaling. These findings provide new evidence of the potential impact of ZIKV on the mature brain and suggest that infection by neurotropic viruses may act as a novel risk factor for the development of type 2 diabetes in infection-recovered patients.

## Results

**Fig. 1:**
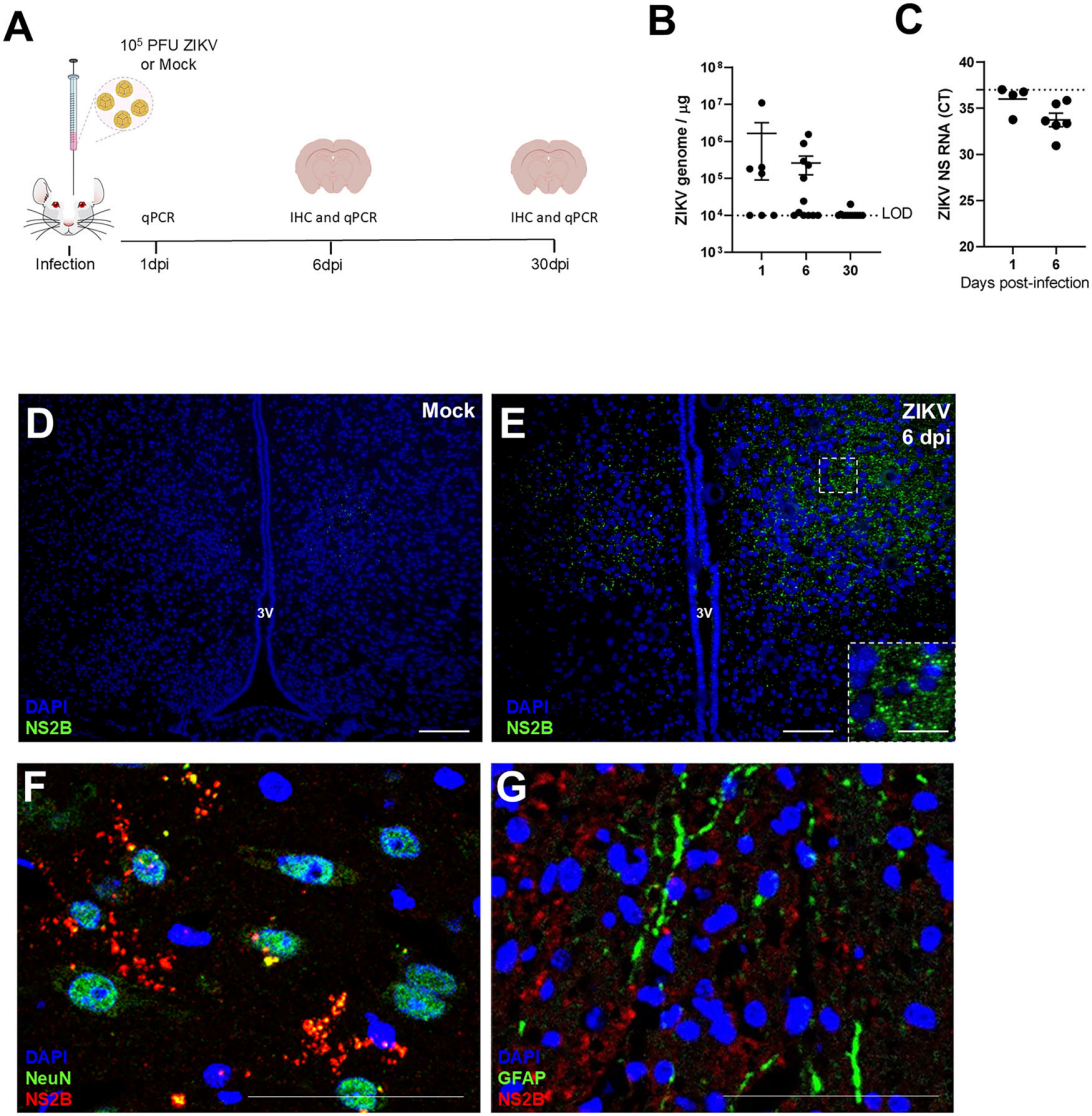
ZIKV infects and replicates in hypothalamic neurons in the adult mouse brain. **A** Two to three-months old mice received an i.c.v. infusion of 10^5^ PFU of ZIKV or mock medium. Brain samples were collected at 1, 6 or 30 dpi, and processed for qPCR or immunohistochemistry. **A**, **B** ZIKV genome copies (**B**; *n* = 7, 12, and 13 for 1, 6 and 30 dpi, respectively). Each symbol represents a different experimental subject. Data are expressed as means ± SEM. **C** ZIKV negative RNA strand (*n* = 4 and 6 for 1, and 6 dpi, respectively) measured in the hypothalamus of mice. **D**, **E** Representative images of immunofluorescence labeling ZIKV (NS2B protein, green) in mock-infused (**D**) and ZIKV infected mice (**E**) at 6 dpi. Scale bar = 200 μm, inset scale bar = 50 μm. **F**, **G** Representative double immunofluorescence images labeling for ZIKV (NS2B protein, red) and NeuN (**F**; NeuN in green) or ZIKV and GFAP (**G**; GFAP in green) in the lateral region of the hypothalamus of ZIKV-infected mice at 6 dpi (*n* = 4). Scale bars in F and G = 400 μm. 3 V=third ventricle.

We have previously shown that ZIKV preferentially targets specific regions of the adult brain, including cortex, hippocampus, striatum, and hypothalamus. Here we investigated the potential impact of central ZIKV infection on hypothalamic function and the regulation of glucose and insulin metabolism in adult mice. We initially confirmed that ZIKV reaches the hypothalamus after systemic infection in type I Interferon-deficient SvA129 (IFNAR^-/-^) mice (10^3^ PFU, i.v.; Suppl. Figure 1A), suggesting that this brain region is targeted when the virus infiltrates into CNS. We then aimed to determine whether the hypothalamus serves as a site of viral replication and, to this end, Swiss mice received an intracerebroventricular (i.c.v.) injection of 10^5^ PFU of ZIKV and hypothalamic samples were collected at 1, 6 and 30 days post-infection (dpi; Fig. 1A). ZIKV RNA quantification by qPCR showed that viral input was detectable at 1 dpi and gradually decreased at 6 and 30 dpi (Fig. 1B). ZIKV negative RNA strand was detected by qPCR in all samples at 1 and 6 dpi (Fig. 1C), suggesting that a sustained, yet likely low-grade viral amplification occurs in the hypothalamus following infection.

Further confirming viral presence in this brain region, immunohistochemistry targeting the serine-protease viral protein NS2B showed significant immunostaining in the hypothalamus of infected mice at 6 dpi (Fig. 1D, E). Double immunolabeling for ZIKV NS2B protein and either neuronal or astrocytic markers showed that NeuN-positive cells were the primary targets of ZIKV in the hypothalamus of mice (Fig. 1F), while no co-localization with glial fibrillary acidic protein (GFAP)-positive cells was detected (Fig. 1G).

**Fig. 2:**
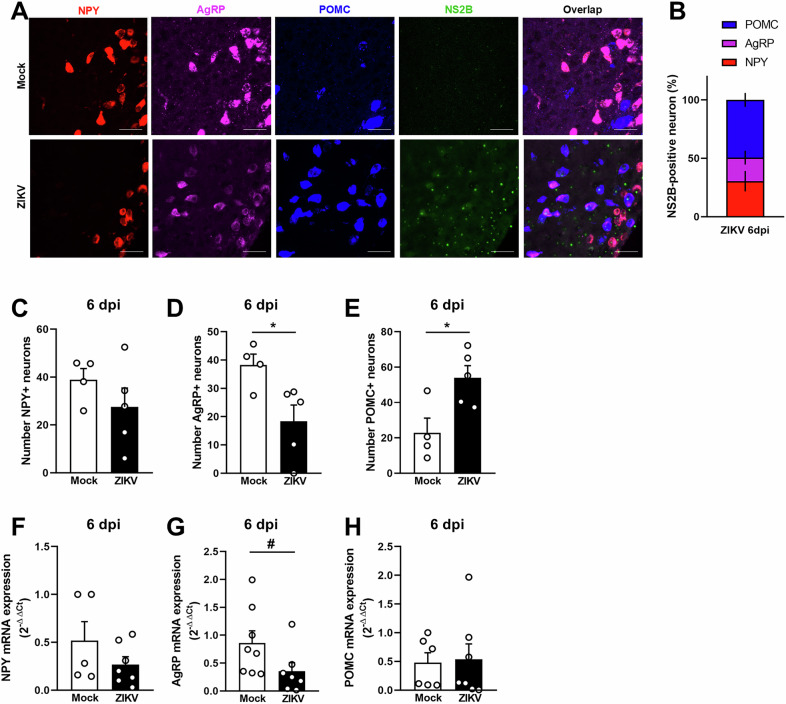
ZIKV targets POMC^+^ cells in the adult mouse brain. Two to three-months old mice received an i.c.v. infusion of 10^5^ PFU of ZIKV or mock medium. **A** Representative images of RNAScope targeting NPY (red), AgRP (pink) and POMC (blue) and immunofluorescence for ZIKV (NS2B protein, green) in mock-infused and ZIKV-infected mice at 6 dpi. Scale bar = 400 μm. (*n* = 4 mock, 5 ZIKV) (**B**) Bar graph represents the percentage of NS2B-positive neurons in each hypothalamic neuron population. **C**–**E** Bar graphs represent the number of NPY^+^ neurons (**C**; Student’s *t* -test. t = 1.152, *p* = 0.2970), AgRP^+^ neurons (**D**; Student’s *t* -test. t = 2.710, *p = 0.0302) and POMC^+^ neurons (**E**; Student’s *t*-test. t = 2.943, *p = 0.0216) revealed by RNAScope in the hypothalamus of mock-infused or ZIKV-infected mice at 6 dpi. **F**–**H** Bar graphs represent NPY (**F**; *n* = 5 mock, 7 ZIKV. Student’s *t*-test t = 1.302, p = 0.2223), AgRP (**G**; *n* = 8 mock, 7 ZIKV. Student’s *t*-test t = 1.864, #p = 0.0850) and POMC (**H**; *n* = 6 mock, 7 ZIKV. Student’s *t*-test t = 0.1716, *p* = 0.8669) mRNA levels measured in the hypothalamus of mock-infused or ZIKV-infected mice at 6 dpi. Data are expressed as mean ± SEM.

The hypothalamus is a complex collection of nuclei expressing different combinations of neuropeptides, which play relevant roles in regulating diverse body functions. For the control of glucose metabolism and brain homeostasis the most important neuropeptides are AgRP, POMC and NPY [18–20]. We then aimed to determine whether one or more populations of hypothalamic neurons expressing these neuropeptides were more susceptible to ZIKV infection. We found that most ZIKV-infected cells in the hypothalamus (~50%) were POMC-positive neurons, while the remaining cells were either AgRP- or NPY- expressing cells (Fig. 2A, B). Also, infection caused no change in the number of NPY-positive neurons (Fig. 2C), we found a significant decrease in AgRP-positive neurons, while POMC-positive cells were significantly increased in the hypothalamus of ZIKV-infected mice compared to control (Fig. 2D, E). We further analyzed the expression of these neuropeptides in the hypothalamus of mice and found that expression of AgRP, NPY and POMC were comparable between ZIKV-infected and mock-injected mice at 6 dpi (Fig. 2F–H). Notably, despite ZIKV neuroinvasion, caspase-1 immunolabeling (Suppl. Figure 1B, C, C’) and TUNEL assay (Suppl. Figure 1G) showed no significant cell death in the hypothalamus of infected mice at 6 dpi. Additionally, the number of hypothalamic NeuN-positive cells (Suppl. Figure 1F–L) as well as the total number of cells (DAPI + , Suppl. Figure 1F–K, M) were comparable between mock-injected and ZIKV-infected mice at this experimental timepoint. Altogether, these findings suggest that changes in hypothalamic cell populations were more likely driven by metabolic alterations rather than cell loss.

We then investigated the potential consequences of ZIKV presence in the hypothalamus of mice, hypothesizing that it could interfere with glucose metabolism and uptake in the brain or peripheral tissues. To assess this, microPET scans were performed immediately before infection and at 6 dpi (Suppl. Figure 2A). ZIKV infection was not associated with significant changes in brain glucose consumption, not only in the hypothalamus but across all analyzed brain regions (Suppl. Figure 2B, C). Given the extensive gliosis caused by ZIKV in mice, it is possible that the overall glucose uptake remains unchanged due to compensatory contributions from glial cells [2]. We further analyzed whether ZIKV hypothalamic infection had consequences at the peripheral level. A significant increase in serum insulin levels was found in ZIKV-infected mice at 6 dpi compared to control (Suppl. Figure 3A), even though this was not associated with changes in basal glucose levels (Suppl. Figure 3B) suggesting that some degree of peripheral insulin resistance occurs during acute stages of infection. Serum triglyceride levels were comparable between mock-injected and ZIKV-infected mice at 6 dpi (Suppl. Figure 3C).

**Fig. 3:**
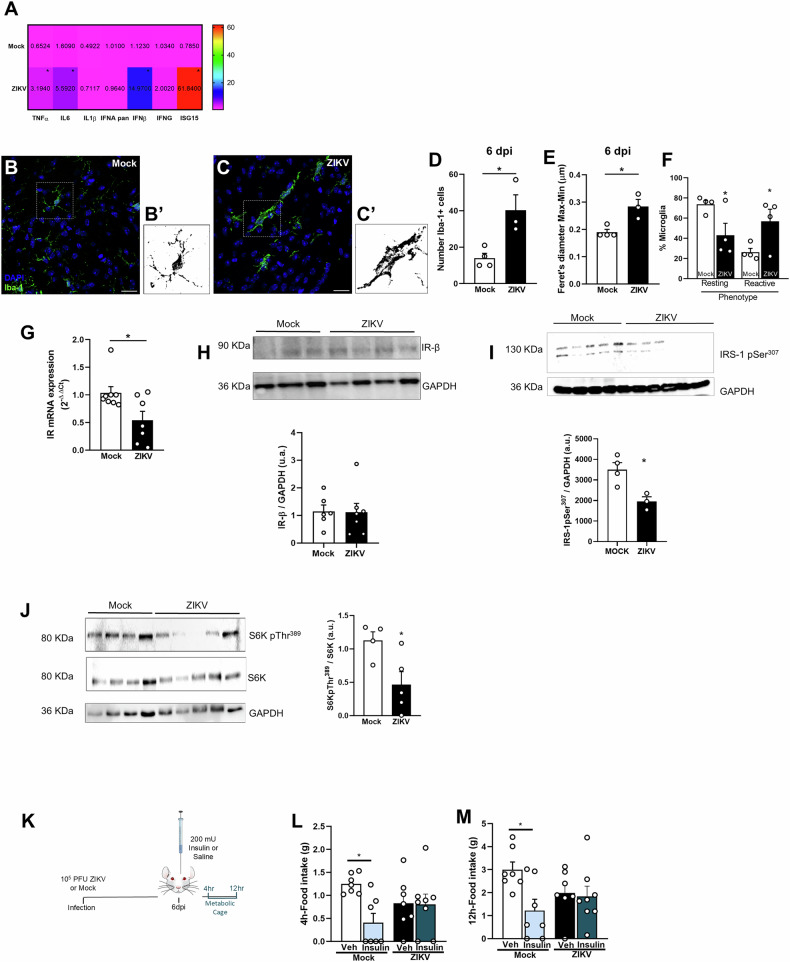
ZIKV neuroinvasion causes hypothalamic inflammation, microglial activation and insulin resistance in mice. Two to three-months old mice received an i.c.v. infusion of 10^5^ PFU of ZIKV or mock medium. **A** Heat Map generated using relative gene expression of TNFα (*n* = 8/group. Student’s *t*-test t = 2.307,* *p* = 0,0381), IL-6 (*n* = 6 mock, 8 ZIKV. Student’s *t*-test t = 1.974, *p* = 0.0700), IL-1β (*n* = 6 mock, 7 ZIKV. Student’s *t*-test t = 0.5307, p = 0.6062), pan IFNA (*n* = 5/group. Student’s *t*-test t = 0.3479, *p* = 0.7369), IFNβ (*n* = 5/group. Student’s *t-*test t = 5.800, *p = 0.0004), IFNg (*n* = 5/group. Student’s *t*-test t = 1.142, *p* = 0.2865), ISG15 (*n* = 5/group. Student’s *t*-test t = 4.652, *p = 0.0023) in the hypothalamus of mice at 6 dpi. **B**, **C** Representative images of Iba immunoreactivity (green) in the lateral region of the hypothalamus of mock-infused (**B**) or ZIKV-infected mice (**C**) at 6 dpi. Scale bar, 25 μm; inset scale bar, 10 μm. **B**’, **C**’ Individual cells highlighted in dashed-line rectangles in (**B**, **C**) were transformed into binary composites and are shown on the right of each representative image. **D**–**F** Bar graphs represent the number of Iba-1+ cells (**D**; *n* = 4 mock, 3 ZIKV, Student’s *t*-test, t = 3.425, *p = 0.0187), Feret’s diameter of Iba-1+ cells (**E**; *n* = 10-15 cells per mouse per mouse, Student’s *t-*test, t = 3.784, *p* = *0.0128)*, and the percentage of Iba-1+ cells with a resting or activated phenotype (**F***; n* = 4/group, Student’s *t*-test,*p = 0.048417 for resting microglia and *p = 0.048423 for reactive microglia) in the hypothalamus of mock-infused or ZIKV-infected mice at 6 dpi. **G** IR mRNA levels in the hypothalamus of mice at 6 dpi (Student’s *t-*test, t = 2.569, *p = 0,0233). **H**–**J** Western blot quantification of IR (**H**; *n* = 6 mock, 7 ZIKV, Student’s *t*-test, t = 0.09129, *p* = 0.9289), phosphorylated IRS-1Ser307 (**I**; *n* = 6 mock, 7 ZIKV, Student’s *t*-test, t = 4.980, *p = 0.005), phosphorylated S6K^Thr389^ (**J**; *n* = 4 mock, 5 ZIKV, Student’s *t*-test t = 2.683, *p = 0.0314) in the hypothalamus of mock-infused and ZIKV-infected mice at 6 dpi; GAPDH was used as a loading control. Representative images from Western blot experiments were always run on the same gels but sometimes in noncontiguous lanes. **J** phosphorylated S6K/S6K t = 2.683, *p = 0.0314. **K** Mice were injected i.c.v. with mock medium or ZIKV and after 6 days received 200mU of insulin or saline as control. Food intake was measured in the metabolic cage after 4 (**L**) and 12 h (**M**; *n* = 7 Mock + Veh, 7 Mock + Insulin, 7 ZIKV + PBS, 8 ZIKV + Insulin, *p = 0,0336, one-way ANOVA followed by Tukey’s post hoc). Data are representative of two independent experiments with similar results. Data are expressed as mean ± SEM. Each symbol represents a different experimental subject.

Landmark studies have shown that hypothalamic inflammation is a central event in conditions of both central and peripheral insulin resistance [21]. We then investigated the extent of hypothalamic inflammation in ZIKV-infected mice and found that, at 6 dpi, several cytokines, including TNF-α, IL6, and IFNβ were upregulated compared to mock-injected animals, as well as the interferon-response molecule ISG-15 (Fig. 3A). In addition, immunolabeling against the ionized calcium-binding adapter molecule 1 (Iba-1) revealed an increased number of microglial/macrophage cells in the hypothalamus of ZIKV-infected mice (Fig. 3B–D). This was accompanied by an increase in the Feret’s diameter of Iba-1-positive cells, indicating that the cells acquired a more amoeboid phenotype (Fig. 3E). Morphology-based classification of Iba-1-positive cells in the hypothalamus further confirmed a decrease in resting-state microglia and an increase in reactive microglial cells in the hypothalamus of infected mice compared to mock-injected animals (Fig. 3F).

We further investigated whether ZIKV-induced hypothalamic inflammation disrupts insulin signaling in mice. At 6 dpi, hypothalamic insulin receptor mRNA levels were decreased in ZIKV-infected mice compared to mock-injected animals (Fig. 3G); however, at the protein level the amount of IR-β was comparable between infected and control groups at this time point (Fig. 3H). The first step in intracellular insulin signaling is the phosphorylation of IRS-1, which modulates downstream signaling. Notably, IRS-1 phosphorylation at Ser307, a key regulatory site that promotes insulin sensitivity [22], was significantly reduced in the hypothalamus of ZIKV-infected mice compared to controls (Fig. 3I). Additionally, phosphorylation of S6 kinase (S6K) at Thr389, a key downstream target of mTORC1 involved in insulin signaling and protein synthesis regulation [23], was significantly reduced in ZIKV-infected mice (Fig. 3J), suggesting impaired activation of this pathway. Central administration of exogenous insulin classically induces a short-term decrease in food intake [24, 25], and the absence of this insulin-triggered ingestion is strong evidence of hypothalamic insulin resistance. To assess whether ZIKV-infected mice exhibited reduced anorexigenic response to centrally administered insulin, food intake was measured in metabolic cages at 6 dpi (Fig. 3K). While a significant decrease in food intake was induced by insulin in mock-injected mice at 4 and 12 h, ZIKV-infected mice were resistant to the effect of insulin on food intake (Fig. 3L, M).

**Fig. 4:**
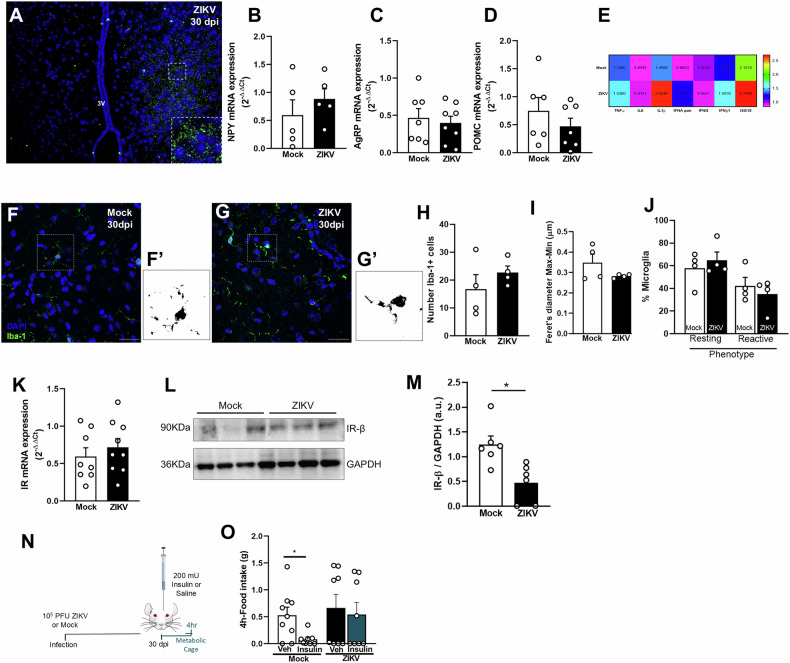
Hypothalamic insulin resistance, but not hypothalamic inflammation and microglial activation, persist beyond acute stages of infection in mice. **A** Representative image of immunofluorescence labeling for ZIKV (NS2B protein, green) in ZIKV infected mice at 30 dpi. Scale bar = 200 μm, inset scale bar = 50 μm. **B–D** Bar graphs represent NPY (**B**; *n* = 5/group, Student’s *t*-test t = 0.8756, p = 0.4068), AgRP (**C**; *n* = 7 mock, 8 ZIKV, Student’s *t*-test t = 0.4565, p = 0.6556) and POMC (**D**; *n* = 6 mock, 7 ZIKV, Student’s *t-*test t = 1.020, p = 0.3296) mRNA levels measured in the hypothalamus of mock-infused or ZIKV-infected mice at 30 dpi. Data are expressed as mean ± SEM. **E** Heat Map generated using relative gene expression of TNFα (*n* = 9/group, Student’s *t*-test t = 0.2754, *p* = 0.7866), IL-6 (*n* = 5 mock, 4 ZIKV, Student’s *t*-test t = 0.1850, p = 0.8593), IL-1β (*n* = 9 mock, 8 ZIKV, Student’s *t*-test t = 1.752, *p* = 0.1001), pan IFNA (*n* = 4 mock, 3 ZIKV, Student’s *t*-test t = 0.5184, *p* = 0.6943), IFNβ (*n* = 5/group, Student’s t -test t = 0.3830, *p* = 0.717), IFNg (*n* = 4 mock, 3 ZIKV, Student’s *t*-test t = 0.3642, p = 0.7306), ISG15 (*n* = 4 mock, 3 ZIKV, Student’s *t*-test t = 0.2735, *p* = 0.7954) in the hypothalamus of mice at 30 dpi. **F**, **G** Representative images of Iba immunoreactivity (green) in the lateral region of the hypothalamus of mock-infused (**F**) or ZIKV-infected mice (**G**) at 30 dpi. Scale bar, 25 μm; inset scale bar, 10 μm. **F’**, **G’** Individual cells highlighted in dashed-line rectangles in (**F,** **G**) were transformed into binary composites and are shown on the right of each representative image. **H**–**J** Bar graphs represent the number of Iba-1+ cells (**H**; *n* = 4/group, Student’s *t*-test, t = 1.050, p = 0.3340), Feret’s diameter of Iba-1+ cells (**I**; *n* = 4/group, 10-15 cells per mouse, Student’s *t-*test, t = 1.513, *p* = 0.1811), and the percentage of Iba-1+ cells with a resting or activated phenotype (**J**; *n* = 4/group, Student’s *t*-test) in the hypothalamus of mock-infused or ZIKV-infected mice 30 dpi. **K** IR mRNA levels in the hypothalamus of mice at 30 dpi (Student’s *t* test, t = 0.7381, *p* = 0.4718). **L**, **M** Representative Western Blot image (**L**) and quantification (**M**) of IR (*n* = 6/group, Student’s *t*-test, t = 3.293, *p = 0.0081) protein levels in the hypothalamus of mock-infused and ZIKV-infected mice at 30 dpi; GAPDH was used as a loading control. Representative images from Western blot experiments were always run on the same gels but sometimes in noncontiguous lanes. **N**, **O** For determination of insulin-induced anorexic effect, mice were injected i.c.v. with mock medium or ZIKV and after 30 days received 200 mU of insulin or saline as control. Food intake was measured in the metabolic cage after 4 h (**O**; *n* = 9 mock+Veh, mock+insulin, ZIKV + Veh, ZIKV + Insulin, *p = 0,0986, one-way ANOVA followed by Tukey’s post hoc). Data are representative of two independent experiments with similar results. Data are expressed as mean ± SEM. Each symbol represents a different experimental subject.

We next investigated whether the effects of ZIKV infection persisted beyond the early phase of viral replication. Immunolabeling for the viral NS2B protein revealed its continued presence in the hypothalamus at 30 dpi (Fig. 4A), suggesting that, although ZIKV genomic material is gradually cleared after the peak of infection (see Fig. 1B), viral proteins may persist in the hypothalamus. To determine whether ZIKV-induced alterations in neuropeptide expression persisted, we analyzed the expression of POMC, AgRP, and NPY, three key hypothalamic neuropeptides involved in glucose homeostasis. At 30 dpi, no significant differences in mRNA levels of these neuropeptides were observed between ZIKV-infected and mock-injected mice (Fig. 4B–D), indicating a return to baseline levels. Additionally, we assessed whether ZIKV-associated hypothalamic inflammation remained at this later time point. Proinflammatory cytokine expression returned to control levels by 30 dpi (Fig. 4E), indicating resolution of the acute inflammatory response. Similarly, no significant differences were observed in the number of Iba-1+ cells, Feret’s diameter, or microglial phenotype in the hypothalamus of ZIKV-infected and mock-injected mice at 30 dpi (Fig. 4F–J), indicating that the transient neuroinflammatory response seen at 6 dpi had resolved by 30 dpi. Furthermore, caspase-1 immunolabeling (Suppl. Figure 4A, B), quantification of NeuN-positive cells (Suppl. Figure 4C–I) and total cells per area (DAPI + , Suppl. Figure 4C–H, J) indicate no significant cell death at 30 dpi. However, while IR mRNA levels in the hypothalamus ZIKV-infected mice were comparable to control animals at 30 dpi (Fig. 4K), protein levels of this receptor were significantly decreased in the hypothalamus of infected mice compared to mock-injected animals (Fig. 4L, M). Additionally, as observed at the peak of viral replication, infected mice failed to exhibit insulin-induced anorexigenic behavior, indicating persistent hypothalamic insulin resistance at 30 dpi (Fig. 4N, O). Altogether, these findings suggest that ZIKV CNS infection is accompanied by transient hypothalamic inflammation, which may induce a lasting disruption of insulin signaling, potentially contributing to long-term metabolic dysfunction.

## Discussion

This study provides novel evidence that Zika virus (ZIKV) infection leads to persistent hypothalamic insulin resistance in adult mice, even after the resolution of acute inflammation. Our findings demonstrate that ZIKV invades the hypothalamus, triggering a robust inflammatory response that disrupts insulin signaling and alters the expression of neuropeptides critically involved in glucose homeostasis. Although inflammatory markers return to basal levels by 30 days post-infection, hypothalamic insulin resistance persists, suggesting long-term metabolic dysfunction. These findings highlight the hypothalamus as a target of ZIKV neuroinvasion, implicating viral infections as potential risk factors for metabolic disorders, such as obesity and type 2 diabetes.

Our data show that ZIKV reaches and replicates in the hypothalamus, as shown by the presence of ZIKV positive-RNA strand. Although viral replication does not persist past acute stages of infection, viral proteins can still be detected in the hypothalamus at 30 dpi. Notably, ZIKV exhibits strong hypothalamic neurotropism, primarily infecting NeuN-positive neurons, while no co-localization was observed in GFAP-positive astrocytes. These findings are consistent with previous studies demonstrating that ZIKV can replicate in the adult brain, including in mature neurons of both humans and mice, leading to synaptic dysfunction, neuroinflammation, and cognitive impairment [2]. Moreover, in developing animals, ZIKV has been shown to infect hypothalamic neuroendocrine cells, disrupting the hypothalamic-pituitary axis and leading to long-term growth and metabolic impairments [10]. Additionally, a recent study demonstrated that postnatal ZIKV infection results in early viral RNA detection in the hypothalamus for up to 7 days post-infection, leading to persistent hormonal deficiencies along the hypothalamic-pituitary-gonadal axis, which in turn disrupts testicular development, sperm quality, and social behaviors [11]. These alterations suggest that ZIKV infection may have significant effects beyond its acute phase, disrupting neuroendocrine homeostasis and potentially leading to long-term physiological dysfunction

The hypothalamus is a key regulator of energy metabolism, controlling glucose homeostasis through neuropeptides such as AgRP, POMC, and NPY [26]. We here showed that ZIKV preferentially infects POMC-positive neurons in the hypothalamus, while AgRP- and NPY-expressing neurons are affected at a smaller scale. Infection leads to a significant reduction in AgRP-positive neurons and an increase in POMC-positive neurons, without altering the number of NPY-positive neurons. Despite these changes, expression analysis at 6 dpi reveals only a trend toward decreased AgRP expression, while POMC and NPY expression remain unchanged. Notably, we did not detect caspase-1 activation in the hypothalamus, suggesting that these alterations are associated with metabolic and functional modifications rather than significant cell death.

We and others have previously demonstrated that ZIKV infection induces significant neuroinflammation in both developing and mature brains [1, 2, 27–29]. Hypothalamic inflammation has been strongly linked to insulin resistance in metabolic disorders [30, 31]. At 6 dpi, the infection induced a robust upregulation of inflammatory cytokines, including TNF-α, IL-6, and IFNβ, together with upregulation of the interferon-stimulated gene ISG-15 in the hypothalamus of mice. Concomitantly, microglial activation was observed, with an increased number of reactive Iba-1+ cells displaying an amoeboid morphology, characteristic of pro-inflammatory microglial activation [32]. These inflammatory changes are known to interfere with insulin signaling pathways, and previous studies have established that hypothalamic inflammation contributes to insulin resistance in metabolic disorders [33, 34].

In models of hypothalamic insulin resistance, transient inflammation induced by a high-fat diet, lipopolysaccharide (LPS) exposure, or cytokine infusion has been shown to cause persistent disruptions in insulin signaling, ultimately contributing to metabolic dysfunction [35, 36]. Our findings indicate that ZIKV infection impairs hypothalamic insulin signaling through multiple mechanisms. At 6 dpi, IR mRNA levels were reduced, along with IRS-1 phosphorylation at Ser307, a key regulatory event for insulin signaling [22] Additionally, S6 kinase (S6K) phosphorylation at Thr389, a critical downstream target of the mTORC1 pathway [23], was also reduced, suggesting impaired insulin-mediated signaling. Similar disturbances in insulin signaling have been reported during acute stages of infection by several viruses including influenza, SARS-CoV-2, HIV, hepatitis C virus (HCV), and dengue virus (DENV), another mosquito-borne flavivirus associated with metabolic alterations and inflammation-driven insulin resistance [37–40]. Notably, we show that ZIKV-infected mice exhibited resistance to the anorexigenic effects of central insulin at both 6 and 30 dpi [24]. While IR protein levels were significantly reduced at 30 dpi and viral proteins persisted in the hypothalamus, neuropeptide levels returned to baseline, and both cytokine upregulation and microglial activation subsided, supporting the idea that ZIKV infection leads to sustained hypothalamic insulin signaling impairment through additional mechanisms. However, unlike other viruses, ZIKV-induced hypothalamic insulin resistance endures beyond the resolution of acute inflammation, suggesting that the virus leaves a persistent footprint on hypothalamic metabolic control. It remains to be established whether this effect is caused by its strong neurotropism and/or potential viral latency in the hypothalamus.

Our findings establish a direct link between ZIKV neuroinvasion and persistent hypothalamic insulin resistance, demonstrating that viral infections can drive long-term metabolic consequences even after the acute phase resolves. We propose that early neuroinflammation and neuronal metabolic reprogramming underlie this persistent impairment. Given that hypothalamic insulin resistance is a hallmark of obesity and type 2 diabetes, these findings suggest that ZIKV infection may contribute to physiological alterations that result in increased risk of metabolic disorders. Understanding these mechanisms could aid the development of strategies to mitigate the lasting metabolic consequences of viral neuroinvasion.

## Methods

### Animals

Male Swiss and C57/Bl6 mice were used in our study. Swiss mice were obtained from our animal facility two to three-months-old at the beginning of experiments. C57/Bl6 mice used in μPET brain imaging experiments were obtained from the Center of Experimental Biological Models (Pontifícia Universidade Católica do Rio Grande do Sul).

Animals were randomly housed in groups of five per cage with free access to food and water, under a 12 h light/dark cycle, with controlled temperature and humidity. All procedures followed the “Principles of Laboratory Animal Care” (US National Institute of Health) and were approved by the Institutional Animal Care and Use Committee of the Federal University of Rio de Janeiro (protocols #126/2018 and #A15/21-126-18).

### Virus

Zika virus was isolated from a febrile case in the state of Pernambuco, Brazil (gene bank KX197192). C6/36 cells were infected at a multiplicity of infection (MOI) of 0.01, in non-supplemented L15 culture medium (Leibovitz’s medium), at a temperature of 28 °C for 1 hour. After this period, culture medium was removed and the cells maintained in L-15 medium supplemented with 5% fetal bovine serum (FBS) at 28 °C for 7 days. The culture supernatant was clarified by centrifugation at 2.4 x g for 10 min, then aliquoted and stored at −80 °C. Plaque lysis assay was performed using VERO cells to determine the titer of viral stocks. These cells were grown on 24-well plates at 37 °C and an atmosphere with 5% CO_2_, and were infected with 200 µl of the serial dilutions (10^−^¹ to 10^−^^6^) of the virus in D-MEM high glucose, and incubated for 1 h in the same culture environment. Then, 1 ml of high D-MEM medium with 1.5% carboxymethylcellulose (CMC, Sigma, CA), 1% FBS and 1% penicillin/streptomycin (Invitrogen) was added to each well. After 5 days, the medium was removed from all wells, cells were fixed with 10% formaldehyde (Vetec, Sigma, CA) and stained with dye solution (20% Ethanol, 1% Crystal Violet and H_2_O) for 30 min, thus allowing visualization and counting of the plaque forming units (PFU). In control groups, the same volume of virus-free C6/36 cells-conditioned medium form (mock) was used.

### Viral infection

For the intracerebroventricular infusion into the lateral ventricle (i.c.v.), animals were anesthetized with 2.5% isoflurane (Cristália, Brazil) using a vaporizer system (Normwell, MA) and were gently restrained only during the injection procedure. As previously described, a 2.5 mm long needle was unilaterally inserted 1 mm to the right of midline point equidistant from each eye and parallel to a line drawn through the anterior base of the eye [2, 24]. Three microliters of a solution containing 10^5^ PFU of ZIKV or mock medium (supernatant of virus-free cell cultures) were slowly infused using a Hamilton syringe. Mice that showed any signs of misplaced injections or brain hemorrhage were excluded from further analysis. In one experiment, type I Interferon-deficient SvA129 (IFNAR^-/-^) mice (obtained from our institutional breeding facility) were anesthetized as described above and were infected with 10³ PFU ZIKV i.v,. via retro-orbital injection.

### RNA extraction and qPCR

Tissue RNA extraction was performed using a tissue grinder and TRIzol (Invitrogen, ThermoFisher Scientific, MA) according to manufacturer’s instructions. RNA purity was determined by absorbance ratios of 260/280 and 260/230 nm. Only samples with absorbance ratios >1.8 and no signs of RNA degradation were used. Two micrograms of total RNA were used for cDNA synthesis using the High-Capacity cDNA Reverse Transcription Kit (ThermoFisher Scientific, MA). To quantify the mRNA of inflammatory genes, cDNA samples were amplified using SYBR Green Master Mix kit (ThermoFisher) and specific primers (sequences are shown on Table 1), were run in a QuantStudio 5 PCR System (Applied Biosystems, Foster City, CA).

**Table 1:** Primer sequences used for qPCR reactions.

Target Gene	*Forward* primers	*Reverse* primers
β-Actin	5’- GCC CTG AGG CTC TTT TCC AG-3’	5’-TGC CAC AGG ATT CCA TAC CC-3’
AgRP	5’-ATG CTG ACT GCA ATG TTG CTG-3’	5’-CAG ACT TAG ACC TGG GAA CTC T-3’
IFNα pan	5’-CCT GAG AGA GAA GAA ACA CAG C	5’-GAG GAA GAC AGG GCT CTC C-3’
IFNβ	5’-CACAGCCCTCTCCATCAACTA-3’	5’-CATTTCCGAATGTTCGTCCT-3’
IFNγ	5’-AGCAACAGCAAGGCGAAAA-3’	5’-CTGGACCTGTGGGTTGTTGA-3’
IL-1β	5’-GTA ATG AAA GAC GGC ACA CC-3’	5’- ATT AGA AAC AGT CCA GCC CA-3’
IL-6	5’-TTC TTG GGA CTG ATG CTG GTG-3’	5’-CAG AAT TGC CAT TGC ACA ACT C-3’
IR	5’-AAA TGC AGG AAC TCT CGG AAG CCT-3’	5’-ACC TTC GAG GAT TTG GCA GAC CTT-3’
ISG15	5′-AAC TGC AGC GAG CCT CTG A-3’	5-′CAC CTT CTT CTT AAG CGT GTC TAC AG-3’
NPY	5’- ATG CTA GGT AAC AAG CGA ATG G-3’	5’-TGT CGC AGA GCG GAG TAG TAT-3’
POMC	5’- ATG CCG AGA TTC TGC TAC AGT-3’	5’- TCC AGC GAG AGG TCG AGT TT-3’
TNF-α	5’- CCC TCA CAC TCA GAT CAT CTT CT-3’	5’- GCT ACG ACG TGG GCT ACA G-3’

For viral quantification, analyses were performed on an Applied Biosystems 7500 RT-PCR system using the TaqMan Mix kit (ThermoFisher Scientific Inc, MA). Primers used for ZIKV RNA amplification were: forward 5’-CCG CTG CCC AAC ACA AG-3’; reverse 5’-CCA CTA ACG TTC TTT TGC AGA CAT-3’; Reporter 5 ‘- / 56-FAM / AGC CTA CCT / ZEN / TGA CAA GCA ATC AGA CAC TCA A / 3IABkFQ / -3’ (Integrated DNA Technologies) as described by [41, 42]. The ZIKV RNA copy number was determined using an amplification standard curve from 10^9^ to 10^1^ copies of a synthetic and reversed transcribed ZIKV RNA oligonucleotide corresponding to the amplified fragment (ZIKV synthetic RNA sequence: 5′-rCrGrG rArCrA rGrCrC rGrCrU rGrCrC rCrArA rCrArC rArArG rGrUrG rArArG rCrCrU rArCrC rUrUrG rArCrA rArGrC rArArU rCrArG rArCrA rCrUrC rArArU rArUrG rUrCrU rGrCrA rArArA rGrArA rCrGrU rUrArG rUrGrG rArCrA rGrArG-3′). Lower limits of detection were determined as CT values of 37 or higher, as previously described [41].

To detect ZIKV negative-RNA strand, total RNA was extracted as described above, and cDNA was synthesized using 2 pmol of ZIKV 835 forward primer instead of random primers, RNA sequence 5’-TTGGTCATGATACTGCTGATTGC-3’. Real-time quantitative PCR analysis was performed as described above.

### Protein extraction/SDS-PAGE/Western blot

Six and 30 days-post infection, mice were euthanized by decapitation and the hypothalamus were rapidly dissected, frozen in liquid nitrogen and lyophilized (−50 °C, 24 h, atmospheric pressure 100 Pa; Liotop, L101, Brazil). Total protein from hypothalamic samples was extracted and homogenized with buffer containing 25 mM Tris–HCl, pH 7.5, 150 mMNaCl, 1% P-40 (Invitrogen), 1% sodium deoxycholate, 0.1% SDS,5 mM EDTA, 1% Triton X-100 and phosphatase and protease inhibitor cocktail (Pierce, ThermoFisher Scientific, IL). Protein concentration was determined using the BCA kit (Pierce). Aliquots containing 30-40 µg protein were then diluted in NuPAGE-LDS sample buffer (in mmol/L: 141 Tris-base, 106 Tris-HCl, 50 dithiothreitol, 0.51 EDTA, 0.22 Coomassie Brilliant Blue G-250, 0.175 phenol red; 2% (w/v) lithium dodecylsulfate, 10% glycerol, pH 8.5) and heated for 2 min at 95 °C. 30–40 μg of protein were loaded in precast NuPAGE Novex 4–12% polyacrylamide gradient gels (Invitrogen, ThermoFisher, MA), along with molecular weight standards (Precision Plus from Bio-Rad, CA), and electrophoresis was carried out at 125 V in a 3-(N-morpholino) propanesulfonic acid (MOPS) running buffer (in mmol/L: 50 MOPS, 50 Tris Base, 1 EDTA, 0.1% SDS, pH 7.7) and were electrotransferred to PVDF membrane for 1 h 15 min at 200 mA. Blots were blocked for 1 h with 5% BSA in Tween-Tris buffer solution at room temperature and were incubated overnight at 2 °C with rabbit anti-insulin receptor β (1:500, Cell Signaling, #3020), rabbit anti-IRS1pSer307 (1:500, Cell Signaling #2381), rabbit anti-S6KpThr 389 (1:1,000, Cell Signaling, #9205), rabbit anti-S6K (1:1,000, Cell Signaling #9202), mouse anti-GAPDH (1:2,000, Abcam, #ab9485) diluted in blocking buffer. After overnight incubation with primary antibodies, membranes were incubated with horseradish peroxidase-conjugated secondary antibodies (1:10,000, Abcam, #6721) at room temperature for 1 h. Chemiluminescence was developed using SuperSignal West Femto (Invitrogen) using a ChemiDoc XRS+ (BioRad).

### Determination of serum glucose in mice

Mice were fasted for 6 h and blood samples were collected from a tail incision for blood glucose measurements, using a Accu-Chek^®^ Performa Roche glucose meter and strips. Mice that showed fasting glucose lower than 50 mg/dL were excluded from the study.

### Determination of serum insulin and triglyceride in mice

Mice were fasted for 6 h before being deeply anesthetized (90 mg/kg ketamine and 4.5 mg/kg xylazine, i.p.). After complete loss of reflex, blood samples were collected and kept on ice until serum separation (centrifugation 2000 g for 15 min at 4 °C). Samples were kept at −20 °C and thawed on the day of insulin and triglyceride detection, using Mouse Insulin ELISA kit (Alpco, Salem, NH) and triglyceride enzymatic kit (Intertek, Brazil), following manufacturer’s instruction.

### Immunofluorescence, TUNEL assay and RNAScope

Animals were anesthetized (90 mg/kg ketamine and 4.5 mg/kg xylazine, i.p.) before perfusion with phosphate buffer saline (PBS, 0.1 mol/L, pH 7.4) and buffered 4% paraformaldehyde (PFA) for immunofluorescence, as previously described by [2]. Brain samples were dissected and post-fixed for 24 h in PFA. After fixation, brains were embedded in paraffin (for immunofluorescence and RNAScope).

For immunofluorescence, paraffin-embedded brain tissue sections (3–5 µm) were immersed in xylene for 10 min, rehydrated in absolute ethanol followed by 95% and 70% solutions of ethanol in water. Antigens were reactivated by treatment with 0.01 M citrate buffer for 40 min at 95 °C. Slides were washed in PBS and incubated with primary antibodies (mouse anti-NeuN, 1:1,000, Cell Signaling #94403; rat anti-GFAP, 1:500, Invitrogen #130300; rabbit anti-Iba-1, 1:1000, Wako NCNP24; rabbit anti-NS2B, 1:50, GeneTex #133308, rabbit anti-Caspase-1, 1:1000, Cell Signaling #3866) diluted in PBS containing 3% BSA. Sections were then incubated with Alexa 594- or 488-conjugated secondary antibodies (1:750; Invitrogen) for 1 h at room temperature, washed in PBS and mounted in Prolong Gold Antifade with DAPI (Invitrogen). Slides were imaged on TCS SP8 confocal microscope (Leica, Wetzlar, Germany) at 200x magnification. Microglia/macrophages (Iba-1-positive cells) were imaged on a TCS SP8 confocal microscope (Leica, Wetzlar, Germany) at 630x magnification. Three independent images were used for analysis. Each image acquired was a z-stack of 12–16 sections (0.33 µm depth).

*Fluorescent Multiplex detection with RNAScope* was performed with Fluorescent Detection kit v2. (cat 323270, Advance Cell Diagnostics, CA) according to the manufacturer’s protocol for formalin-fixed paraffin-embedded tissue. Detection was performed by Opal Dye 520 for *Npy*, Opal Dye 570 for *AgRP* and Opal 620 for *POMC*. Following in situ hybridization, sections were processed for immunofluorescence. Briefly, following the blocking step with 5% normal goat serum (NGS, Vector Laboratories, CA) for 2 hours at room temperature, post-hybridization slides were incubated with NS2B antibody (1:500, GeneTex, #133308) in blocking solution overnight at 4 °C. After washing with PBS, slides were incubated with corresponding AlexaFluor 488 secondary antibodies (1:500, Abcam, ab150077) for 2 h at RT. Images were acquired on a Nikon A1RHD+ confocal microscope (Nikon, Melville, NY).

*TUNEL assay*. Quantification of apoptotic cells was performed by TUNEL assay method using DeadEnd Fluorometric TUNEL System following manufacturer’s instructions (Promega Corporation, Madison, WI). Images were obtained on a Sight DS-5M-L1 digital camera (Nikon, Melville, NY) connected to an Eclipse 50i light microscope (Nikon). At least three sections from each animal were analyzed, cortex to the same animal was used as a control.

### ^18^F-Fluorodeoxyglucose (^18^F-FDG) μPET Brain Imaging

Male C57/Bl6 mice were used for in vivo μPET image acquisition, following the administration of ^18^F-FDG. The images were acquired at two different timepoints: immediately before ZIKV infection (baseline) and at 6 days after the injection of 10^5^ PFU of ZIKV into the lateral ventricle (*n* = 8). Images were obtained in a Triumph μPET (LabPET-4, TriFoil Imaging, CA). For imaging, animals received an intraperitoneal injection of 250 μCi of ^18^F-FDG and were placed in their home cages for a 40 min period of awake radiotracer uptake. Following the uptake period, each mouse was anesthetized with isoflurane, and medical oxygen (3%–4% for induction, and 2%–3% for maintenance) and positioned in a head-first prone position with the field of view centered in the animal’s head. Ten-minute list mode static images were acquired. Data were reconstructed using the Maximum Likelihood Estimation Method (MLEM-3D) algorithm. The reconstructed images were spatially normalized into an ^18^F-FDG template using PMOD v3.8 and the FUSEIT Toolbox. The standardized uptake value (SUV) was calculated for the whole brain and each individual region of interest. To correct for weight variations, the SUV ratio (SUVr) of each individual brain region was determined by dividing the SUV value of the region by the whole brain SUV [42, 43]. All animals were kept on a heating pad at 36 °C throughout the experiment and fasted overnight before the scan to increase the uptake of ^18^F-FDG by the brain.

### Metabolic cage experiments

*Swiss* mice were submitted to i.c.v. injection of three microliters of a solution containing 10^5^ PFU of ZIKV or mock medium. Five and 29 days after injection mice were individually placed in metabolic cages (3600M011, Tecniplast, Italy) with free access to food and water, and allowed to acclimate. After 24 h (corresponding to 6 or 30 dpi), at the beginning of the dark cycle, mice received an i.c.v. injection of either PBS or human recombinant insulin (Humulin, 200 mU in 3 µL) and, after having recovered from anesthesia, were placed in the metabolic cage. The amount of powdered chow eaten was determined per animal at 4 and 12 h after insulin injection and food intake was determined by the difference between chow given to mice immediately after injection and the weight of remaining chow 12 h after.

### Statistical analysis

Graphs and statistical analysis were performed using GraphPad Prism 8 (GraphPad, CA). Data are expressed as means ± SEM and the identification of outliers was evaluated using the ROUT test. D’Agostino-Pearson normality test used to assess the Gaussian distribution of the data. Student’s *t* tests were performed to compare two groups with comparable variances and the *t* test with Welch’s correction was applied when comparing two groups with unequal variances. One-way ANOVA with appropriate post-hoc analysis or Student’s t tests were performed to compare different groups (as indicated in Figure Legends). P values were calculated for each experiment and p values smaller than 0.05 were considered statistically significant.

## Supplementary information

Supplemental Figure Legends

Suppl. Fig. 1

Suppl. Fig. 2

Suppl. Fig. 3

Suppl. Fig. 4

Suppl. Fig. 5

## Data Availability

The datasets generated during and/or analysed during the current study are available from the corresponding author on reasonable request.
