# Supplementary material for: Central Zika virus infection causes hypothalamic inflammation and persistent insulin resistance in adult mice
Source: Cell Death Dis. 2025 Oct 13;16(1):722. doi: 10.1038/s41419-025-08046-5 (PMC12518652; doi:10.1038/s41419-025-08046-5)
Supplement: Supplementary file 1 — Supplemental Figure Legends [file 41419_2025_8046_MOESM1_ESM.docx]

**Supplemental Figure 1. ZIKV central infection does not cause significant caspase-1 activation in the hypothalamus of adult mice. (A)** SVA mice received an i.v. infusion of 10³ PFU of ZIKV or equal volume of mock medium. ZIKV genome copies were measured by qPCR in the hypothalamus of infected mice at 6 days post-infection (dpi). *n* = 4, each symbol represents a different experimental subject. (**B-D)** Representative images of immunofluorescence labeling for Caspase-1 (green) in the lateral region of the hypothalamus of Swiss mock-infused (**B**) or ZIKV-infected mice (**C**,**C’**) at 6 dpi. (**D**) Representative image of Caspase-1 immunolabeling the cortex of ZIKV-infected mice at 6 dpi. (**E-E’**) Representative images of TUNEL labelling (green) and DAPI (blue) in the lateral region of the hypothalamus **(E)** and the hippocampus (**E’**) of Swiss ZIKV-infected mice at 6dpi. Representative images of immunofluorescence labeling for NeuN (red) in the lateral region of the hypothalamus of Swiss mock-infused mice (**F-H**) and ZIKV-infected mice at 6 dpi (**I-K**). Bar graphs represent the number of NeuN positive cells (**L**) and DAPI positive cells (**M**) in the lateral region of the hypothalamus of ZIKV-infected mice at 6dpi (**L**; n= 4 mock and 5 ZIKV. Student’s t test. t=1.550, p=0.1599, **M**; n=5 Mock and ZIKV. Student’s t test. t=1.125, p=0.2977).

**Supplemental Figure 2. ZIKV central infection does not cause net changes in brain glucose consumption.** (**A**) ^18^F-FDG uptake was determined before (baseline) and 6 days after i.c.v. infusion of 10^5^ PFU of ZIKV or mock medium. (**B**) Representative coronal (left), sagittal (middle) and transverse (right) micro-PET images of cerebral ^18^F-FDG uptake before (top images) and after ZIKV infection (bottom images). (**C**) Index of brain glucose metabolism in µPET brain imaging (*n*=6/group, One-way ANOVA). Data are expressed as mean ±SEM. HPT= hypothalamus; HPC= hippocampus; STR=striatum; CTX= cortex; CLB=cerebellum; THL= thalamus.

**Supplemental Figure 3.** **ZIKV central infection causes increase in serum insulin but does not cause alteration in blood glucose and serum triglycerides.** Two-three-months old mice received an i.c.v infusion of 10^5^ PFU of ZIKV or mock medium. Bar graphs represent serum insulin that was measured through ELISA assay after 6h fasting (**A**; *n= 5*/group, Student’s *t test*. t=3.876, *p=0.0047). (**B**) Bar graphs represent blood glucose after 6h fasting *(n= 7/*group. Student’s *t* test t=1.427, p=0.1791). Bar graphs represent serum triglycerides after 6h fasting (**C**, *n=5* Mock and *7* ZIKV. Student’s *t* test, t=1.175, p=0.2672).

**Supplemental Figure 4. ZIKV central infection does not cause significant caspase-1 activation in the hypothalamus of adult mice at later stage. (A-B)** Representative images of immunofluorescence labeling for Caspase-1 (green) in the lateral region of the hypothalamus of mock-infused (**A**) or ZIKV-infected mice (**B**) at 30 dpi. Scale bar: 200 μm; (**C-H**) Representative images of immunofluorescence labeling for NeuN (red) in the lateral region of the hypothalamus of Swiss mock-infused mice (**C-E**) and ZIKV-infected mice (**F-H**) at 30dpi. Bar graphs represent the number of NeuN positive cells **(I)**, and DAPI positive cells and (**J**) in the lateral region of the hypothalamus of ZIKV-infected mice at 6dpi (**I**; n=3 Mock and ZIKV. Student’s t test. t=0.3599, p=0.7371; **J**, n= 4 mock and ZIKV. Student’s t test. t=0.2378, p=0.8200).

**Supplemental Figure 5.** The original western blots reported in this study
