# Supplementary figures and images for: Central Zika virus infection causes hypothalamic inflammation and persistent insulin resistance in adult mice
Source: Cell Death Dis. 2025 Oct 13;16(1):722. doi: 10.1038/s41419-025-08046-5 (PMC12518652; doi:10.1038/s41419-025-08046-5)

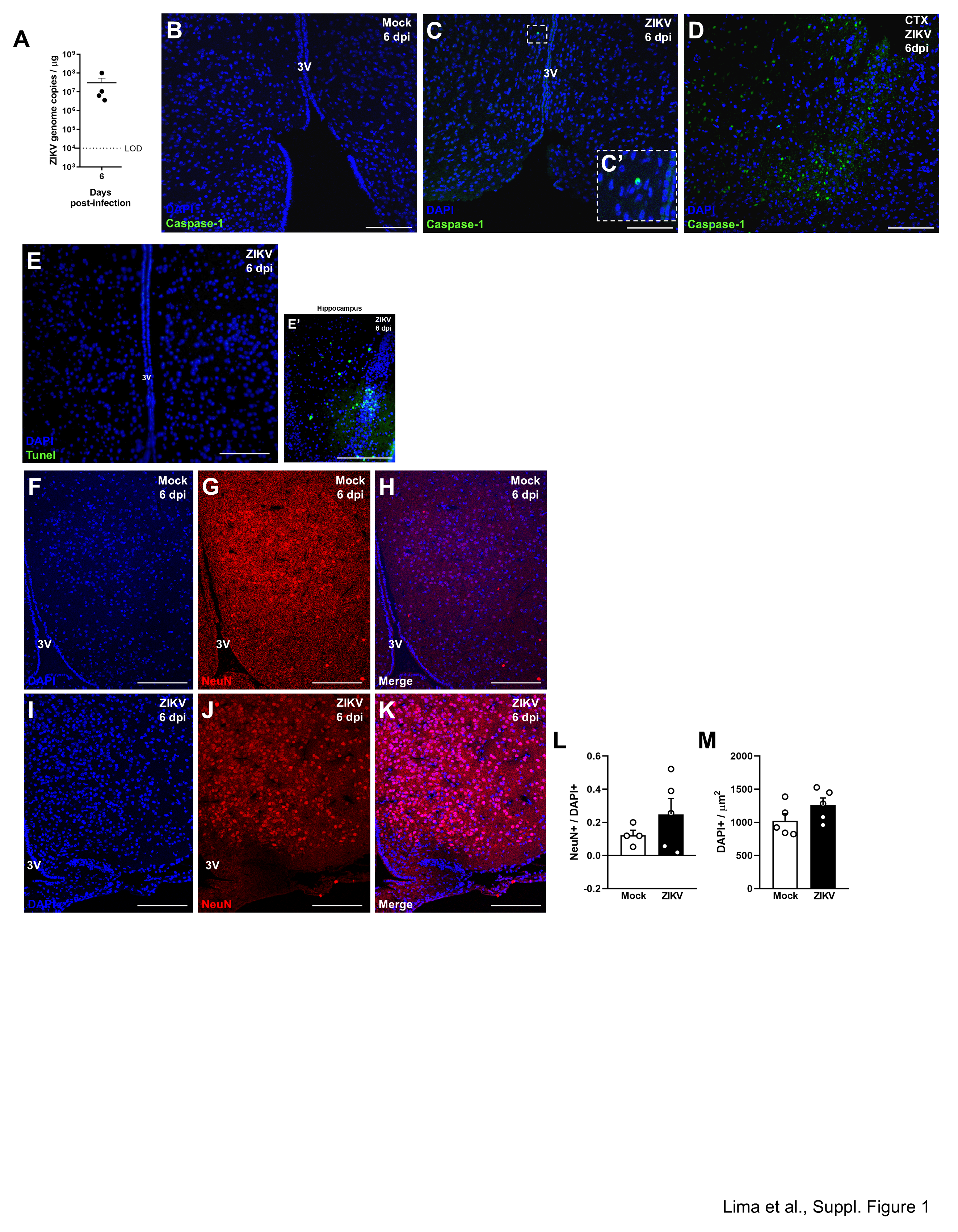

Supplement: Supplementary file 2 — Suppl. Fig. 1 [file 41419_2025_8046_MOESM2_ESM.png]

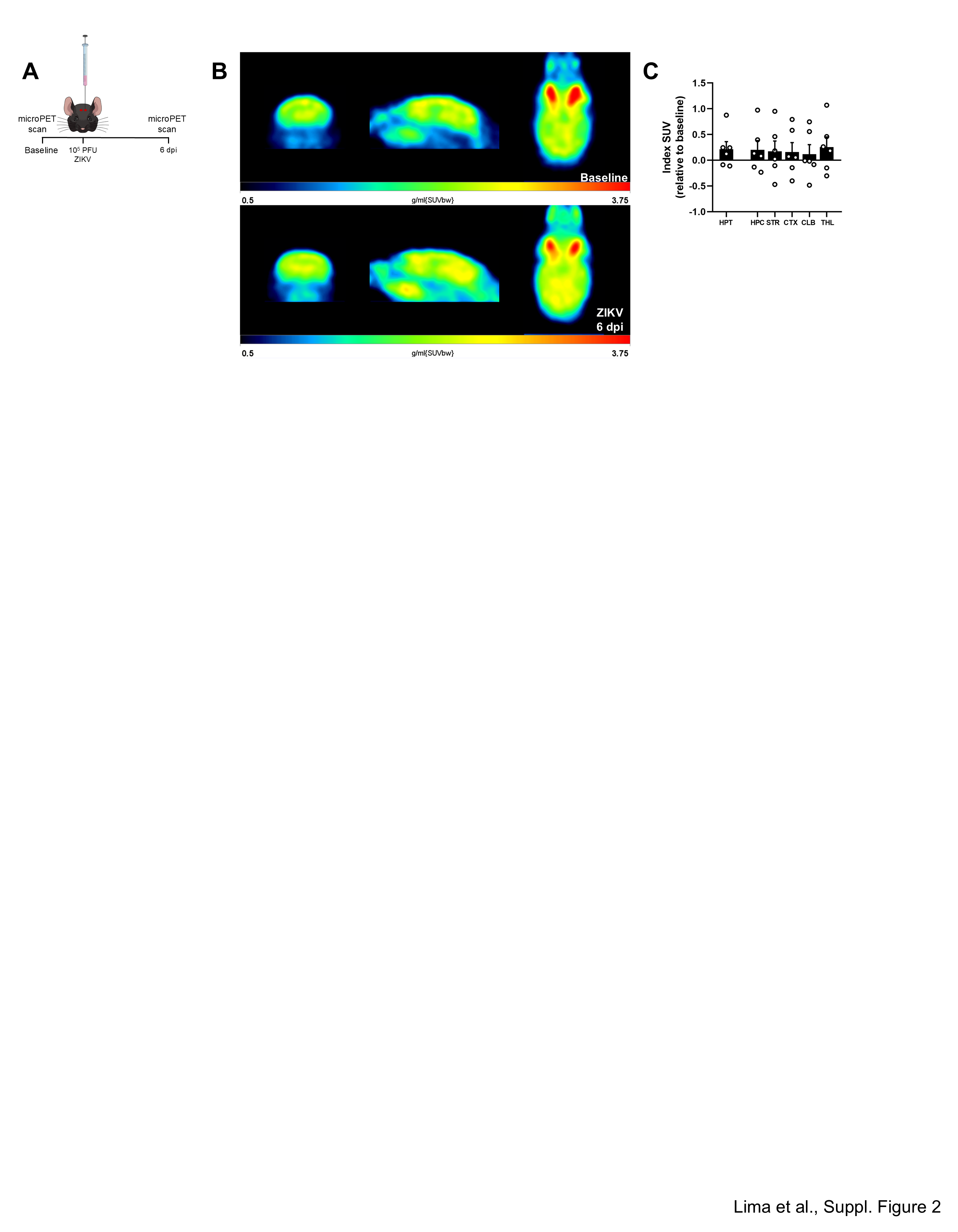

Supplement: Supplementary file 3 — Suppl. Fig. 2 [file 41419_2025_8046_MOESM3_ESM.png]

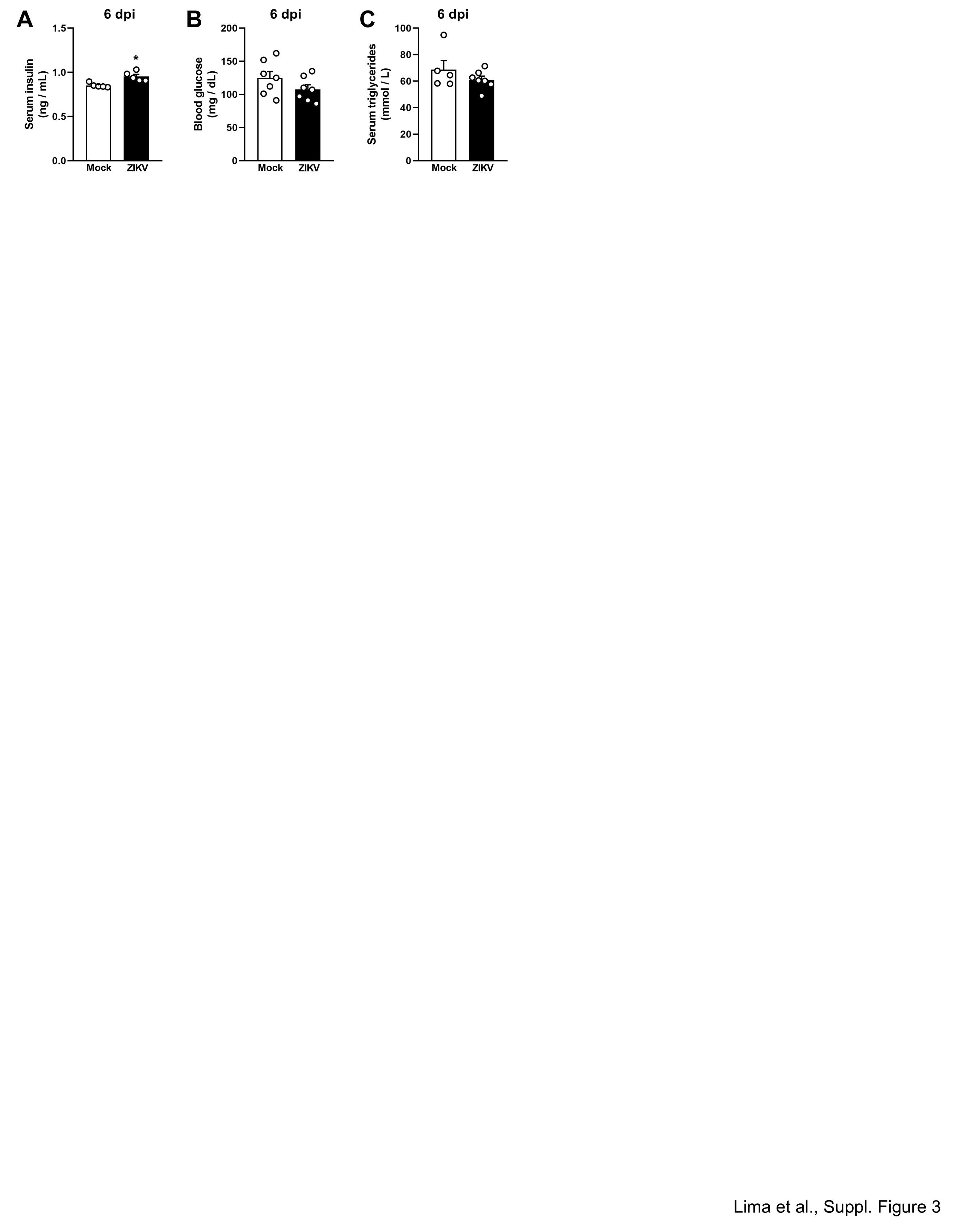

Supplement: Supplementary file 4 — Suppl. Fig. 3 [file 41419_2025_8046_MOESM4_ESM.png]

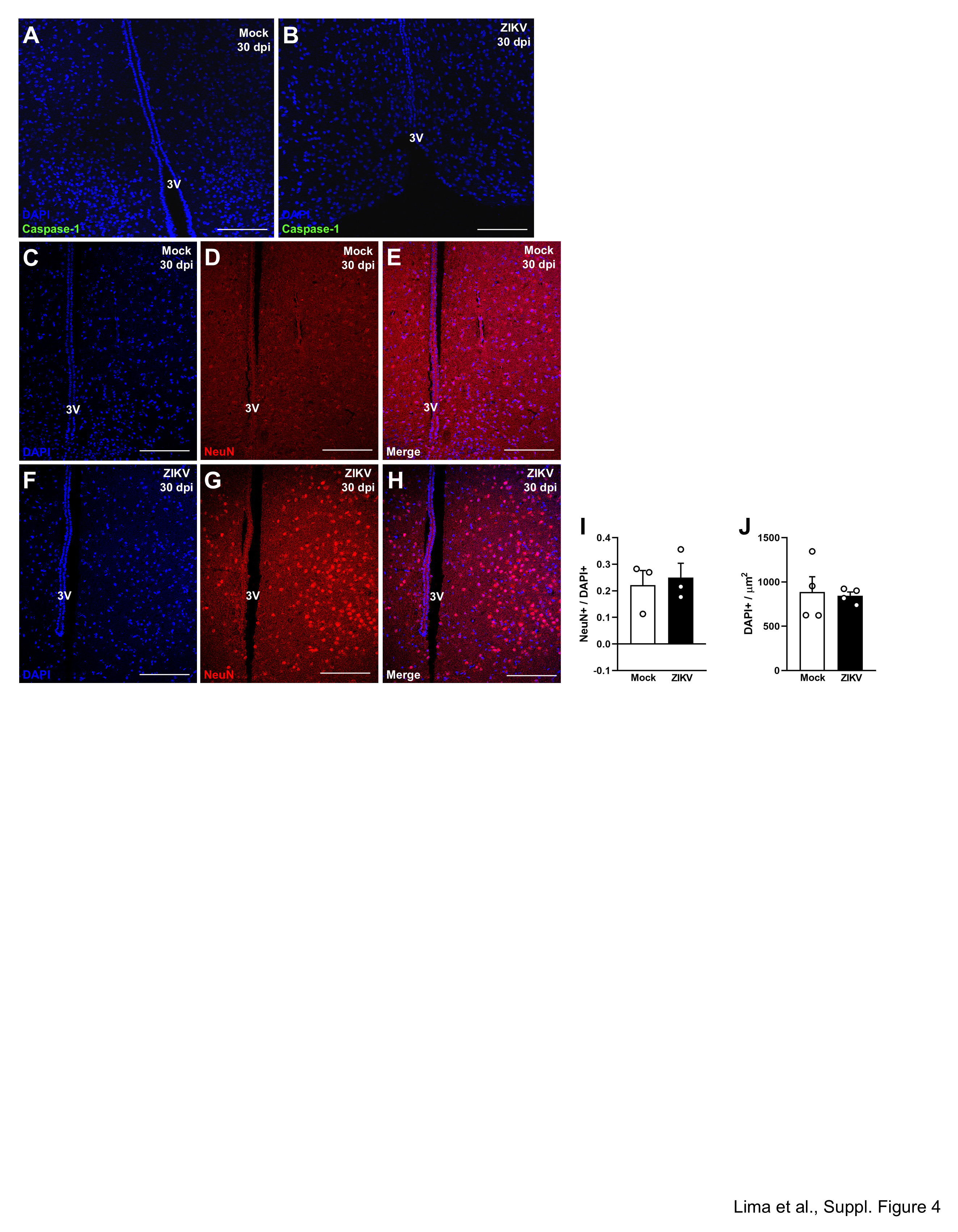

Supplement: Supplementary file 5 — Suppl. Fig. 4 [file 41419_2025_8046_MOESM5_ESM.png]

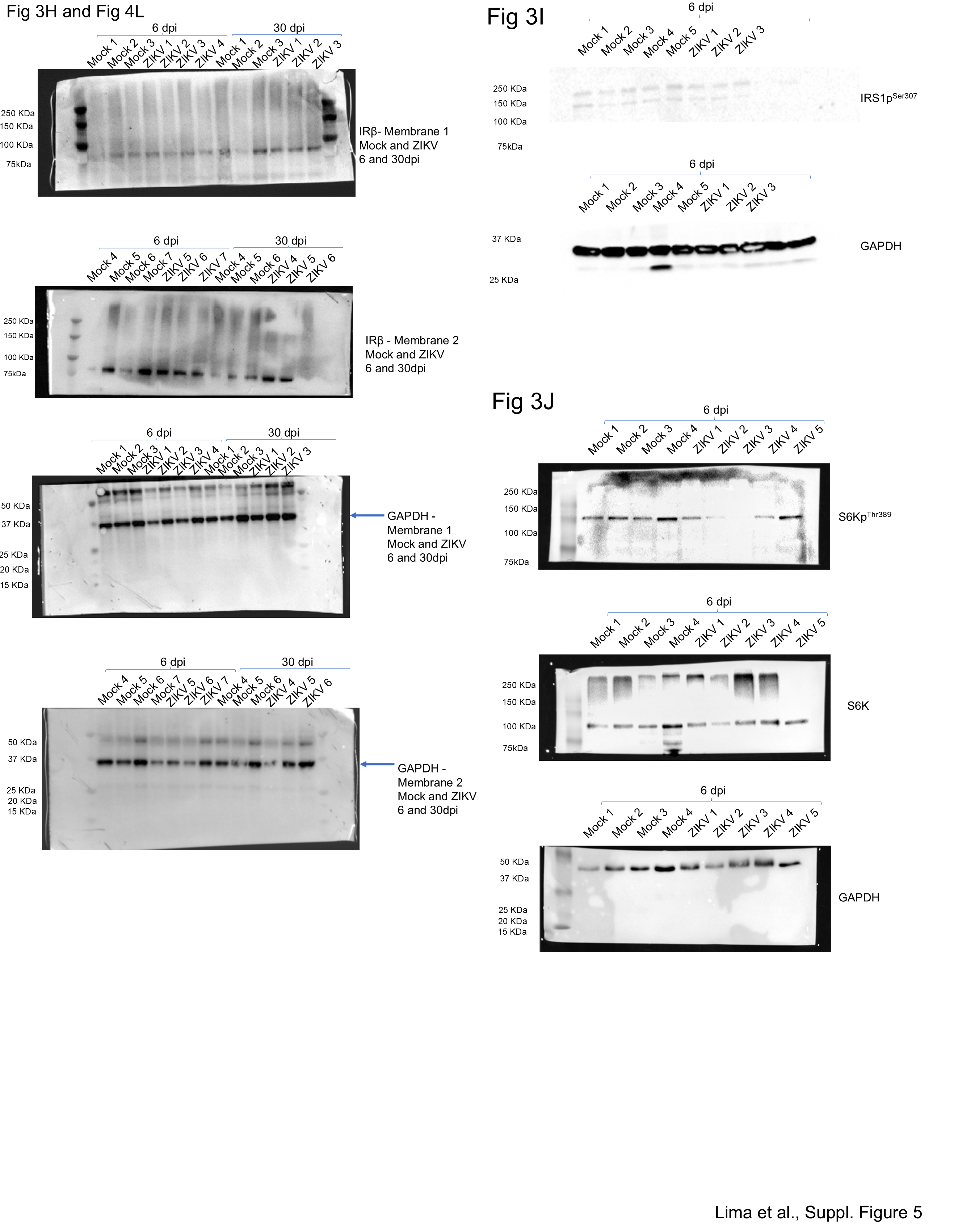

Supplement: Supplementary file 6 — Suppl. Fig. 5 [file 41419_2025_8046_MOESM6_ESM.png]
